# Stewardship of Antibiotics for Multidrug-Resistant Gram-Negative Bacteria

**DOI:** 10.3390/antibiotics9040206

**Published:** 2020-04-24

**Authors:** Daniele Roberto Giacobbe, Ilias Karaiskos

**Affiliations:** 1 Infectious Diseases Unit, Ospedale Policlinico San Martino—IRCCS, 16132 Genoa, Italy; 2 Department of Health Sciences, University of Genoa, 16132 Genoa, Italy; 3 Department of Internal Medicine-Infectious Diseases, Hygeia General Hospital, 15123 Athens, Greece; ikaraiskos@hygeia.gr

Nearly one year ago, we wrote the following introductory note for authors willing to submit their paper to our Special Issue entitled “Stewardship of Antibiotics for Multidrug-Resistant Gram-Negative Bacteria” in Antibiotics: 

“In the last decades, multidrug-resistant Gram-negative bacteria (MDR-GNB) have represented an important threat, for several reasons. Above all, the paucity of dependable therapeutic options, which, until just a few years ago, often relied on potentially nephrotoxic drugs and/or on drugs with possible suboptimal efficacy. Recently, some much awaited novel agents have become available, restoring our ability to effectively counteract some perilous infections due to these organisms. However, it remains imperative to administer novel agents thoughtfully and in line with antimicrobial stewardship principles, in order to: (i) delay the development of resistance to novel agents; (ii) delay the diffusion of resistance to novel agents, since some cases of inherent or acquired resistance have already been reported. In light of this, our efforts to optimize the use of old agents should also not be discontinued, since they still remain essential for treating infection due to MDR-GNB nonsusceptible to novel agents”.

Reflecting this background and after a rigorous peer review of 16 submissions, as many as 11 papers were accepted for publication. Interestingly, they include both original articles and narrative reviews, and cumulatively address stewardship concerns regarding both old and novel agents employed in the treatment of MDR-GNB infections.

With regard to the antimicrobial stewardship of “old” agents, Atsushi Uda and colleagues depict an antimicrobial stewardship intervention conducted over 5 years in patients with urinary tract infections, resulting in a decrease in the prevalence of inappropriate antibiotic prescriptions (especially in that of inappropriate prescriptions of anti-pseudomonal penicillins) [1]. Despite some important limitations such as the retrospective design and the absence of a control group, their results are consistent with the well-established idea of sparing old but important agents (in the sense that they may remain active against some MDR-GNB) when they are not really necessary. In other words, we certainly need to use novel agents whenever indicated, but this necessity can be reduced by wisely using old agents in order to preserve susceptibility in the long term. This concept is also extensively addressed By Ilias Karaiskos and Helen Giamarellou in their narrative review on the use of carbapenem-sparing agents for extended spectrum β-lactamase (ESBL)-producing *Enterobacterales* [2]. The authors comprehensively discuss a much-debated topic, in which the true positioning of various β-lactams as effective carbapenem-sparing agents is still only partly understood. We thus encourage readers to take a look at their detailed summary [2]. Other articles included in the issue that explore stewardship opportunities for old agents include the one by Márió Gajdács, detailing how to possibly spare colistin (but also novel agents) in patients with urinary tract infections due to carbapenem-resistant *Pseudomonas aeruginosa* [3], and the one by Giacobbe and colleagues, discussing the long-term positive results of an antimicrobial stewardship project aimed at reducing the incidence of infections due to carbapenem-resistant *Klebsiella pneumoniae* in a cardiosurgery department [4]. Finally, in their surveillance study, Mahmoud and colleagues highlight the need for effective stewardship intervention in cancer patients with hospital-acquired urinary infections due to ESBL-producing *Escherichia coli* [5]. 

With regard to novel agents, Vena and colleagues describe a cohort of 41 patients treated with ceftazidime/avibactam for infections due to MDR-GNB other than carbapenem-resistant *Enterobacterales*, reporting high rates of clinical success (90.5%) in a peculiar population that is certainly not the usual target of real-life ceftazidime–avibactam administration (which is correctly reserved for KPC- and OXA-type carbapenemase-producing *Enterobacterales* in most cases), suggesting that its wise use outside the usual scenario may also be appropriate in selected cases [6]. Another novel agent that may remain active against carbapenem-resistant *Pseudomonas aeruginosa* is ceftolozane/tazobactam. While its use in adults has been extensively described in the literature, there are few data about its use in children. In this issue, Ahmed Zikri and Kamal El Masri report the successful treatment with ceftolozane–tazobactam of an immunocompromised pediatric patient with pneumonia due to MDR *Pseudomonas aeruginosa* [7]. Finally, the narrative review by Marianna Criscuolo and Enrico Maria Trecarichi put together the currently available experiences of MDR-GNB infection treated with ceftazidime/avibactam or ceftolozane/tazobactam in patients with hematological malignances [8]. In our opinion, the peculiar interest of this review lies in the fact that, as also noted by the authors, although they are not specifically approved for neutropenic/cancer patients, there is growing interest in using these drugs in this peculiar population, because of the increasing prevalence of infections caused by MDR-GNB reported in some hematological centers [8].

Overall, there are plenty of interesting topics about the stewardship of old and novel agents for MDR-GNB infections that are explored in the articles published in the present Special Issue. In addition, we included two papers by Pillay and colleagues and Oloso and colleagues, which deal with resistance issues in the broiler production chain, reminding us of the importance of adopting a One Health approach in global antimicrobial stewardship interventions [9,10]. Finally, we tried to look to the future with a brief narrative review on the use of machine learning techniques in observational studies of MDR-GNB infections in humans [11]. The field is still in its infancy, but we like to think that it will be an important addition to our concerted antimicrobial stewardship efforts in the future.

In conclusion, we would like to offer our profound thanks to all the authors who actively participated in this Special Issue. We hope *Antibiotics* readers will enjoy reading it and find it useful for their everyday clinical and stewardship practice.
